# Abiraterone for castration-resistant prostate cancer: adherence, survival and hospitalization: Analysis of a medical claims database

**DOI:** 10.1007/s00508-016-1067-9

**Published:** 2016-09-05

**Authors:** Badereddin Mohamad Al-Ali, Gero Kramer, Stephan Madersbacher, Ingrid Berger

**Affiliations:** 1 Department of Urology and Andrology, Kaiser-Franz-Josef Spital, Kundratstrasse 3, 1100 Vienna, Austria; 2 Department of Urology, Medical University of Vienna, Vienna, Austria; 3 Department of Urology, Landesklinikum Wiener Neustadt, Wiener Neustadt, Austria; 4 Department of Urology, Medical University of Graz, Graz, Austria

**Keywords:** Medication adherence, Prostate cancer, Abiraterone acetate, Castration-resistant prostate cancer, Survival

## Abstract

**Objective:**

To analyze the drug adherence rates and overall survival for in patients treated with arbiraterone acetate (AA) for castration-resistant prostate cancer (CRPC).

**Methods:**

The database of the largest insurance company in Austria (Wiener Gebietskrankenkasse) was analyzed. Data on all CRPC patients with at least one prescription of AA between November 2011 and December 2014 in the postchemotherapy setting were collated and compared to the Austrian death and hospital admission statistics. Drug adherence was estimated by the medication possession ratio (MPR).

**Results:**

Data of 270 patients (mean age 73.5 ± 8.9 years) were analyzed. The mean duration of AA treatment was 9.8 months (range 1–38 months). The duration of AA treatment was as follows: 0–2 months 53 patients (19.6 %), 3–5 months 73 patients (28.1 %), 6–10 months 67 patients (24.8 %) and >10 months 97 patients (35.9 %). The median MPR was 100 % and in 241 (89.2 %) the MPR exceeded ≥80 %. The median overall survival (OS) was 11 months. Based on Kaplan-Meier analysis, the 6 month OS was 61 %, 12 month OS 43 %, 18 month OS 35 % and >24 month OS 24 %. The OS was strongly correlated to patient age and the duration of AA treatment. Of all 270 patients, only 19 (7 %) were not hospitalized during their remaining life span and 71 (26.2 %) spent more than 50% of their remaining life span in hospital care.

**Conclusion:**

The OS was shorter than in phase III trials and strongly correlated to patient age and the duration of AA treatment. The high mortality rate within the first 6 months of AA treatment in this real-life setting suggests a less stringent patient selection than in a phase III trial.

## Introduction

In the past 15 years oral anticancer medications have become more widely available for the treatment of a variety of cancers and have increasingly been used as an alternative to intravenous (iv) therapy. This trend is also demonstrable in the management of advanced prostate cancer (PC). The introduction of the oral drugs abiraterone acetate (AA) and enzalutamide [1] has revolutionized the management of castration resistant prostate cancer (CRPC). The availability of oral anticancer drugs aligns with the preferences of cancer patients and may help to improve patient quality of life and reduce the time spent in healthcare settings; however, the availability of oral drugs induces the issue of drug compliance or adherence [2, 3]. Both terms define the extent to which patients take medications as prescribed by their healthcare providers. Without adequate adherence, the efficacy of oral drugs might fall below of those administered intravenously. The second threat of an oral drug, particularly if well tolerated such as AA and enzalutamide, is that these drugs might be prescribed too liberally.

Prompted by the paucity of data of both aspects we investigated these issues by analyzing the database of the largest public health insurance in Austria (Wiener Gebietskrankenkasse, WGKK). This database was compared to the Austria death statistics and hospital admission registry. For decades Austria has had a public healthcare system with compulsory state insurance. The high prevalence of CRPC as well as the cost of these drugs further emphasize the economic impact of drug adherence and prescription pattern.

The aim of this study was to evaluate (i) adherence patterns for AA and concomitant prednisone in patients with CRPC in Austria*, *(ii) the overall survival of CRPC receiving AA in a real-life setting and (iii) hospital admission rates in this cohort by using a large administrative healthcare database.

## Material and methods

After receiving institutional board approval from our hospital the prescription database of the largest public insurance company in Austria the WGKK was reviewed. Data from all patients with at least one prescription of AA from November 2011 to December 2014 were extracted, where AA [1] is typically prescribed at 1000 mg daily taken orally with 10 mg oral prednisone. The following data were extracted from this database: age, data of the first AA prescription, number of AA prescriptions, number of hospital admissions and duration of hospital admissions. This database was matched to the Austria death and hospital admission statistics.

Adherence [2, 3] was calculated using the medication possession ratio (MPR), which is the sum of all days of AA supplied within a given period, divided by the total number of days in that period. There is no consensus standard for what constitutes adequate adherence. Some trials consider rates of greater than 80 % to be acceptable [4, 5].

### Statistical analyses

The SPSS 17.0 package for Windows (SPSS, Chicago, IL) was used for statistical analysis and all the values were expressed in terms of means ± SD for the efficacy analysis. Survival time was calculated from the initiation of AA and the date of death. Patients alive were censored at the last known follow-up date. Overall survival (OS) rates were estimated using the Kaplan-Meier method.

## Results

### Patient characteristics and AA prescription pattern

Data of 270 patients with CRPC with at least 1 prescription of AA were analyzed. The mean patient age was 73.5 ± 8.3 years (median 74 years). The age distribution was as follows: ≤60 years 19 patients (7.0 %), 61–70 years 82 patients (30.4 %), 71–80 years 105 patients (38.9 %) and >80 years 66 patients (24.4 %). The mean duration of AA treatment in all patients was 9.5 months (range 1–38 months). The duration of AA treatment was as follows: 0–2 months 53 (19.6 %), 3–5 months 73 (28.1 %), 6–10 months 67 (24.8 %) and >10 months 97 (35.9 %).

### Drug adherence

The mean MPR was 94.8 ± 11.9 with a median value of 100 %. The mean MPR ranged from 40–100 %. The MPR was not dependent on patient age (Table 1). In the age group ≤70 years the mean MPR was 95.7 ± 12.1 as compared to 95 ± 10.7 in those older than 70 years. In 241 (89.3 %) patients the MPR was ≥80 % and the MPR was below the critical value of 80 % only in 29 (10.7 %) patients.

**Table 1:** Medication possession ratio

	Medication possession ratio	
	Mean	Median
Total patients (*n* = 270)	94.8 ± 11.9	100
Age 46–60 (years, *n* = 19)	94.9 ± 12.2	100
Age 61–70 (years, *n* = 80)	95.7 ± 12.1	100
Age 71–80 (years, *n* = 105)	95 ± 10.7	100
Age 81–92 (years, *n* = 66)	93.5 ± 13.6	100

### Survival

**Fig. 1:**
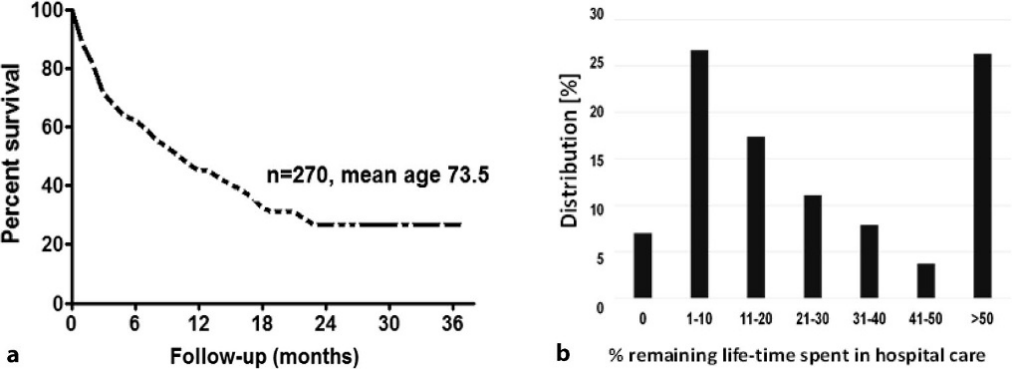
**a** Overall survival of the total study cohort (*n* = 270). **b** Frequency distribution of the remaining life span spent in hospital care

**Fig. 2:**
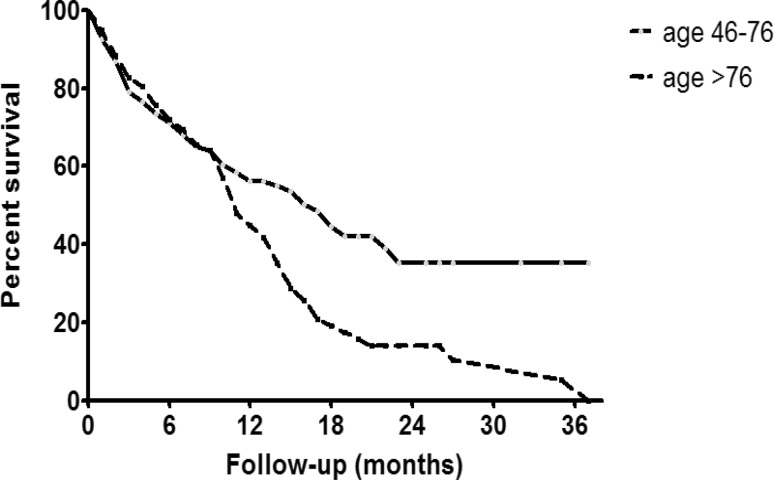
Overall survival stratified by patient age

**Fig. 3:**
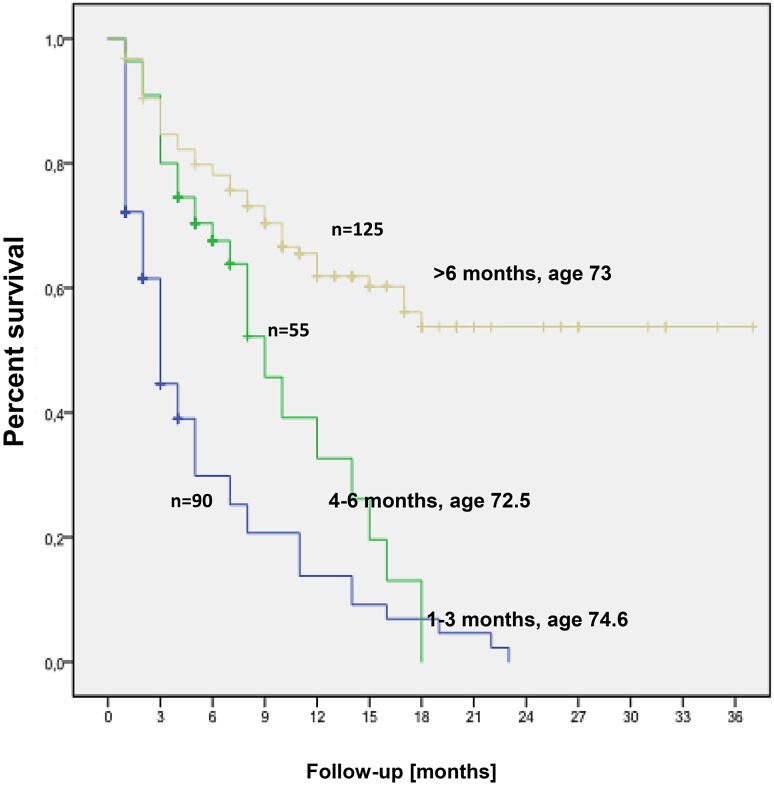
Overall survival stratified by duration of AA treatment

The mean OS was 15.7 ± 1.1 months (median 11 months) with a range of 1–38 months (Fig. 1a). According to Kaplan-Meier analysis, the 6 month OS was 61 %, the 12 month OS 43 %, the 18 month OS 35 % and the 24 month OS 24 %. One quarter of patients survived longer than 2 years (Fig. 1a). Patient age had a profound impact on OS (Fig. 2). At 18 month follow-up, 42 % of the younger age cohort (median age 69.5 years) were still alive as compared to only 19 % in the elderly cohort (median age 83 years) (Fig. 2). It is worth noting that 35 % of all patients in the younger cohort experienced a survival of longer than 2 years. The median OS in the younger cohort was 17 months (mean 19.1 ± 1.4 months) as compared to only 5 months (mean 8.8 ± 1.0 months) in the elderly cohort. Fig. 3 presents the OS depending on the length of AA treatment and OS was also strongly dependent on the duration of AA treatment (Fig. 3).

### Hospital admissions

In Austria the vast majority of chemotherapies for CRPC are given on an outpatient basis in urological or oncological institutions. For reimbursement reasons, these patients are admitted on a day case basis and are included to the hospital admission statistics; therefore, we have deleted all admissions for 24 h or less from further analyses.

The mean length of hospital stay was 4.5 ± 4.8 days (median 2 days). The duration of the hospital admissions was as follows: 1–2 days 55.8 %, 3–4 days 13.4 %, 5–6 days 6.9 %, 6–8 days 7.3 %, 8–10 days 5.5 % and >10 days 11.1 %. The patients in this series spent a considerable time of their remaining life in hospital care. Of all 270 patients only 19 (7 %) were not hospitalized during their remaining life span. On the other end of the spectrum 71 (26.2 %) spent more than 50 % of their remaining life span in hospital care (Fig. 1b).

## Discussion

The principal findings of this Austrian prescription database analysis were (i) high adherence to AA, (ii) high mortality within the first months of AA prescription, (iii) 24 % long-term survivors under AA and (iv) high rate of hospitalization in patients under AA.

Prior to discussion, several pros and cons of our approach need to be discussed. Strengths are: (i) the population-based character of this database. The WGKK is the biggest insurance company in Austria (8.4 million insured persons) and it has 1.2 million members in all socioeconomic classes, (ii) for decades Austria has had a public and equal access healthcare system with compulsory state health insurance company and (iii) long follow-up and complete survival data due to matching with the Austrian death statistics. The main limitation is the lack of any clinical information (e.g. indications, previous chemotherapy, staging and reason for drug discontinuation). Finally, the data on hospital admissions have to be interpreted in the context of the Austrian healthcare system.

Cancer treatment is evolving. Chronic oral administration transfers responsibility from the practitioner to the patient, making adherence an important parameter in reducing the risk of treatment failure. Adherence to medical treatment is a complex and multifaceted issue that can substantially alter the outcome of therapy [6–10].

Outside randomized controlled trials (RCT) few studies have reported on the adherence to AA. Smith et al. [4] analyzed pharmacy claims of the Canadian Saskatchewan Cancer Agency. All patients with at least one AA prescription were eligible and a total of 86 patients were followed for a minimum of 6 months. Optimal drug adherence was achieved in 82.6 % of patients with 79.1 % reaching a MPR of at least 90 %. At 6 months the mean MPR was 89.6 % (median 100 %) and after 12 months 86.6 % (median 99.5 %). Lafeuille et al. [5] studied this issue by analyzing two large-scale US administrative healthcare claims databases. The mean age of the patients was 72.2 years and the mean MPR was 93 % (median 98 %). The mean MPR in our series was 94.8 ± 11.9 (median 100 %) with no relevant impact of patient age in contrary to the study by Grundmark et al. [6]. Taken together, these two studies with a substantial number of analyzed patients indicate that the adherence to AA in a real-life setting is satisfactory with median values exceeding 95 %.

In contrast to the prescription database studies described previously, this current one is unique with respect to the availability of survival data. There is a major concern that survival data generated by RCT or registries do not reflect the real life setting [11–16]. It has been previously shown that eligibility to a chemotherapy protocol represents per se a good prognostic factor [17]. In the pivotal phase III trial of AA in the postchemotherapy setting de Bono et al. [18] reported on a median survival of 14.8 months as compared to 11 months in our series. The median age of our patients, however, was 6 years older compared to the de Bono et al. [18] trial. The maximum follow-up in our study was considerable longer (38 months) as compared to the pivotal phase III trial with 20 months. There are a considerable number of long-term survivors. In our series, 24 % of patients survived longer than 20 months and 37 % in the phase III trial. The early mortality rate, however, was substantially higher in our series. Within the first 6 months of AA treatment 39 % of our patients died as compared to only 15 % in the de Bono et al. phase III trial [18]. These data suggest that patient selection in real life is substantially less stringent than in a phase III trial. In our series, patient age had a relevant impact on overall survival. In patients aged 46–76 years the median survival was 17 months and thus longer than in the pivotal RCT as compared to only 5 months in those older than 76 years. Houede et al. [19] reported on the long-term outcome of the AA temporary authorization for use program in France: 306 patients with a median age of 63 years were analyzed. The overall survival in this cohort after initiation of AA was 14.6 months, almost identical to the RCT and longer than in our series. As expected, OS was correlated to the duration of AA treatment, a similar phenomenon was observed in our series (see Fig. 3).

The third aspect of this study was the analysis of hospital admissions and length of hospitalization after initiation of AA. In our series, these patients spent a considerable time of their remaining life in hospital care. Only 7 % were not hospitalized and one quarter of patients (26.2 %) spent more than 50 % of their remaining life span in hospital care. These data have to be interpreted in the context of the Austrian healthcare system, where admission to hospital care is liberal and free of charge to the patient; furthermore, there is no incentive to discharge patients as early as possible. We could not identify a comparable analysis with CRPC patients under chemotherapy and second-line endocrine therapy in the literature.

According to Svensson et al. [20] patients were on average 2 years older than those in the RCT, which is partly in agreement with our study population, OS in Swedish patients was the same like de Bono et al. [18] (COU-AA-301 trial), in contrary to our results, and the researchers concluded that the treated population and treatment patterns, organization of healthcare, as well as country setting could contribute to differences in outcomes between the clinical trial and the real world treatment, which could be an explanation for our OS results in comparison to the randomized controlled trials.

Outcomes of treatment in clinical practice can differ from outcomes in RCT with regard to the estimated effectiveness and the estimated resource utilization. Discrepancies in patient and physician behavior between the trial and the real world may have an impact on outcome and treatment cost.

In clinical trials patients continued treatment until documented progression while the real world evidence study collected information on progression. There is much interest in confirming whether the efficacy of AA demonstrated within the trial setting is reproducible in routine clinical practice in a non-trial setting and many differences should be taken in consideration between both, such as the selection of patients and ethnic differences.

The OS in the study of Poon et al. [21] of chemotherapy-naive patients was 18.1 months and much shorter than that reported in the COU-AA-302 study (of 34.7 months) Ryan [16] and a higher proportion of elderly patients, which is in agreement with our study.

### Limitations

The main limitation is the lack of any clinical information (e.g. indications, previous chemotherapy, staging and reason for drug discontinuation). Finally, the data on hospital admissions have to be interpreted in the context of the Austrian healthcare system where admission to hospital care is voluntary and free of charge to the patient; furthermore, there is no incentive to discharge patients as early as possible.

## Conclusion

This Austrian prescription database allows some relevant insights into the outcome of patients treated with AA for CRPC in a real life setting. Drug adherence was satisfactory and OS was shorter as compared to the pivotal phase III trial. The high early mortality rate in our series suggests poor patient selection in real life. One quarter of patients experience long-term survival. The hospitalization rate within this cohort was substantial.
